# Different Stages, Different Signals: The Modulating Effect of Cognitive Conflict on Subsequent Processing

**DOI:** 10.1371/journal.pone.0163263

**Published:** 2016-09-16

**Authors:** Fada Pan, Liang Shi, Li Zhang, Qingyun Lu, Song Xue

**Affiliations:** 1 School of Education Science, Nantong University, Nantong, China; 2 School of Public Health, Nantong University, Nantong, China; 3 State Key Laboratory of Cognitive Neuroscience and Learning & IDG/McGovern Institute for Brain Research, Beijing Normal University, Beijing, China; Southwest University, CHINA

## Abstract

The present study used event-related potentials (ERPs) to investigate the function of signals induced by cognitive conflict during the detection stage and the resolution stage of perceptual processing. The study used a combination of the Stroop task and an affective priming task to examine the conflict priming effect when the stimulus onset asynchrony (SOA) was 200 ms or 800 ms. Behavioral results showed that the RTs were shorter for positive targets following congruent primes relative to incongruent primes, and for negative targets following incongruent primes relative to congruent primes when the SOA was 200 ms. ERP results showed that the N2 amplitudes (200–300 ms) for incongruent stimuli were significantly larger than for congruent stimuli in the Stroop task, which indicated a significant conflict effect. Moreover, the N400 amplitudes (500–700 ms) for positive targets after congruent primes were significantly lower than those after incongruent primes when the SOA was 200 ms, which showed a significant negative priming effect. While the SOA was 800 ms, behavioral results showed that the RTs were shorter for positive targets following incongruent primes relative to congruent primes. ERP results showed that the N2 amplitudes (200–300 ms) for incongruent stimuli were significantly larger than for congruent stimuli in the Stroop task, which indicated a significant conflict effect. The N400 amplitudes (1100–1300 ms) for the negative targets after congruent primes were significantly lower than those after incongruent primes when the SOA was 800 ms, which showed a significant positive priming effect. The results demonstrated that the functions of signals induced by cognitive conflict were reversed in two different cognitive processing stages.

## Introduction

Cognitive control refers to the human ability to adjust actions and goals in response to external and internal demands during ongoing information processing [1]. In everyday life, for example, if right-handed persons are required to write or eat with their left hand, they would find it difficult to do and they would have to pay more attention to complete the task. In the laboratory, cognitive control is mainly studied by using a conflict-response paradigm, as conflict is a primer for cognitive control processing [2]. Over the past few decades, studies on control did not just involve “cold” cognition, but involved emotional processing and affective adjustments [3, 4]. Evidences from brain imaging researches have shown that the anterior cingulate cortex (ACC) plays a major role in cognitive-control processing [2].

However, there are two seemingly opposite theories of ACC function. One is the conflict-monitoring theory, which holds that cognitive control is triggered by the detection of response conflicts [2]. The other theory is that the ACC evaluates outcomes, and the role of the ACC is to detect, register, and evaluate performance, with the outcome evaluation being a signal for selecting future action [5, 6, 7]. Recently, Botvinick [3] integrates both two theories and suggests that the ACC’s role is to detect negative signals in general. This opinion is supported by the evidences from brain imaging studies, which show that the ACC is not only activated by conflict and error feedback, but also by social exclusion [8] and experienced or observed pain [9]. However, if it is true, conflict should also be viewed as a negative affective signal.

Dreisbach and Fischer [10] applied a rather straightforward method to study the affective valence of conflict. They used incongruent or congruent Stroop color words as affective primes and positive or negative words as targets, combining a Stroop task with an affective priming paradigm. In the typical affective priming paradigm, a positive or negative appears firstly, followed by a target with certain valence (positive or negative), and participants are required to judge the valence of target without responding to the prime. The typical findings are that positive primes accelerate the judgment of positive targets, whereas negative primes accelerate the judgment of negative targets. As predicted, Dreisbach and Fischer [10] found shorter reaction times (RTs) for negative targets following incongruent primes and longer RTs for positive targets following incongruent primes. It was taken as the first direct evidence for the negative valence of conflict. Recently, data from their laboratory also showed that compared with congruent primes, incongruent primes induced more negative judgments on the following neutral targets [11]. Other theoretical accounts, as well as physiology [12, 13] and psychology findings [14, 15], suggest that conflicts are viewed as negative signals, and have a negative effect on subsequent information processing. In other words, conflicts produce a negative affective signal that induces the negative priming effect.

Shortly afterwards, Fritz and Dreisbach [16] investigated the time course of the negative conflict by controlling the time interval between the onset of the prime stimulus and the onset of the target stimulus (stimulus onset asynchrony, SOA). Their results showed that the priming effect had something to do with SOAs. More specifically, a negative priming effect was found when the SOA was 200 ms or 400 ms. However, it disappeared when the SOA was 800 ms and the priming effect was even reversed.

Schouppe et al. [17] used a combination of Flanker task and affective priming paradigm to study the affective valence of congruency conditions following a successful response. Participants in the affective priming task were instructed to respond to the prime stimulus firstly, and then to judge the valence of target stimulus. They found the priming effect, but it was reversed after a successful response. A relatively positive effect was more likely to be triggered after a correct response on incongruent trials than on congruent trials. In their view, correctly responding to incongruent primes indicated conflict resolution. Thus, when participants were required to judge the targets, the nature of the primes did not have an influence and induce the conflict priming effect.

Hence, the above results about positive prime effect imply that cognitive conflict can induce both negative and positive evaluative signals. The reward value and prediction model (RVPM) of the ACC proposed by Silvetti, Seurinck, and Verguts [18] predicts that once an incongruent trial is correctly solved, it will evoke a positive prediction error signal. That is to say, there will be a shift from a negative signal to a positive signal following conflict resolution (i.e., responding correctly to an incongruent stimulus). In line with this idea, Molapour and Morsella [19] demonstrated that nonsense shapes co-occurring with incongruent Stroop stimuli were preferred over shapes that co-occurred with congruent or neutral Stroop stimuli. Participants in this study were required to respond to each Stroop stimulus.

In summary, there are two opinions about the signal induced by cognitive conflict. Some studies indicate conflicts should be viewed as negative signals that induce a negative priming effect [10, 14], whereas other studies suggest cognitive conflicts can induce both negative and positive signals. Cognitive conflict can induce a positive signal following conflict resolution. Given these results, is there any comprehensive explanation that could integrate these points of view? The answer is yes, as far as I am concerned. Conflicts are perceived as negative signals that induce negative priming effect when they first appear (that is, our brain detects this signal). However, once the conflict processing is completed (that is, it is resolved), the negative influence will weaken and even disappear. Moreover, it may evoke a positive effect on subsequent cognitive processing.

The present study used event-related potentials (ERPs) to confirm such hypothesis, combining Stroop paradigm and affective priming task. Two experiments were designed with different SOAs: 200 ms or 800 ms. The prime stimulus consisted of incongruent and congruent Chinese color characters. The participants were instructed to respond to the target stimuli, but not to respond to the prime stimuli. In general, we expected an interaction between the priming condition and the target’s valence. Specifically, when the SOA was 200 ms that is at the conflict detection stage, we expected there would be a negative priming effect. That meant negative targets after incongruent primes would be evaluated faster than those after congruent primes. However, the effect would be just opposite when the targets were positive. We also expected that N2 amplitudes for incongruent primes would be significantly higher than those for congruent primes, and that the N400 amplitudes for negative targets after incongruent primes would be significantly lower than those after congruent primes, and vice versa. On the other hand, when the SOA was 800 ms that is at the conflict resolution stage, we expected there would be a positive priming effect. That meant positive targets after incongruent primes would be evaluated faster than those after congruent primes. Nevertheless, the effect would be converse when the targets were negative. We also expected the N2 amplitudes for incongruent primes would be significantly higher than those for congruent primes, and that the N400 amplitudes for positive targets after incongruent primes would be significantly lower than those after congruent primes, and vice versa.

## Experiment 1

### Methods

“This study was approved by the Human Ethics Committee of Nantong University. A written informed consent was obtained from all the participants”.

#### Participants

Twenty healthy undergraduate students (10 males, 10 females, with average age of 20.4 years, ranging from 19 to 23) volunteered to participate in the study. All subjects were right-handed, had no mental illness, color blindness or color weakness, and had normal or corrected-to-normal vision. Everyone was given a small gift in return for the participation.

#### Apparatus and stimuli

The primes were the Chinese characters for the colors RED, YELLOW, BLUE, GREEN, and PURPLE, which were printed in red, yellow, blue, green, and purple. If the character and its color were mismatched (such as RED printed in blue), the prime was incongruent; if the character and its color matched (such as RED printed in red), the prime was congruent. The target stimuli were 88 Chinese words that were chosen from the Chinese Affective Words System (CAWS) [20]. The valence ratings of positive words were over 6.90 and the valence ratings of negative words were below 3.08. The prime and target stimuli were all written in 34-point Times New Roman font, and were presented centrally on a light gray background. The experiment was run on an Asus Computer and programmed in E-Prime (Psychology Software Tools).

#### Procedure

A practice Stroop task was conducted before the formal experiment in order to make the participants familiar with Stroop primes, especially with the incongruent primes. This Stroop task consisted of 24 incongruent and congruent trails in random order. Participants were required to judge the ink color of the word, while ignoring its semantic meaning.

The formal experiment was following the above practice task. Each trial started with a red fixation cross for 250 ms, followed by a Stroop prime (incongruent or congruent) for 200 ms. After the Stroop prime, the target word appeared and remained on the screen until a response was made. The next trial started after an inter-trial-interval of 1000 ms.

Participants did not need to respond to the Stroop prime, but just to judge the valence of target word by pressing the “f” key on the keyboard when the target was positive word and pressing the “j” key for the negative target word. The assignment was kept constant, since people have a tendency to associate right with positive and left with negative [21]. Catch trials were included to ensure participants processed the prime stimuli: whenever the primes BLUE, GREEN, YELLOW, or RED were printed in purple, or the prime stimulus was the Chinese character “PURPLE”, participants had to press the space button as quickly as possible instead of judging the following target.

The experiment started with a practice block containing 28 trials to make the participants familiar with the task. The practice block was followed by an experimental block with 176 trials, including 160 target trials and 16 catch trials. In these 160 target trials, there were 80 positive targets preceded by 40 incongruent Stroop primes and by 40 congruent Stroop primes; the remaining 80 trials were negative targets also preceded by 40 incongruent and 40 congruent Stroop primes.

#### ERP recording and analysis

The electroencephalogram (EEG) was recorded on a Brain Products (Germany) system using a 64-channel electrode cap, with the references on the left and right mastoids and a ground electrode on the medial frontal aspect. The electro-oculogram (EOG) was recorded from the outer canthi of the right eye and below the left eye. All the electrode impedances were kept below 20 KΩ. The continuous EEG signals were recorded at sampling rates of 500 Hz. EEG data were recorded simultaneously with the collection of the behavioral data (accuracy and reaction time) and were subjected to off-line analysis.

The recorded data were analyzed using Brain Vision Analyzer 2.0. Eye blinks, eye movements, and other artifacts were removed from the averaging. The EEG activity during the 200 ms prior to stimulus onset served as the baseline. All epochs exceeding ±100 μV were automatically excluded from further processing. Averages were computed for each condition and subject separately.

The international standard 10–20 system was used to place the electrodes. We analyzed the N2 component of the Stroop effect with 10 electrode sites in the frontal-central areas: FCz, Cz, FC3, FC4, FC5, FC6, C3, C4, C5, and C6. The ERPs waveforms were time-locked to the onset of Stroop prime and the average epoch was 1000 ms. The following nine electrode sites in the central-parietal areas were selected to analyze the affective priming effect on the N400 component: Cz, CPz, Pz, C3, C4, CP3, CP4, P3, and P4. The ERPs waveforms were also time-locked to the onset of Stroop prime, thus the average epoch was respectively extended to 1200 ms. That meant we selected Stroop prime as stimulus onset and the EEG activity during the 200 ms prior to stimulus onset served as the baseline in the analysis of N2 and N400. The time windows of N2 and N400 should be, respectively, 200–300 ms and 500–700 ms.

### Results

#### Behavioral results

**Fig 1:**
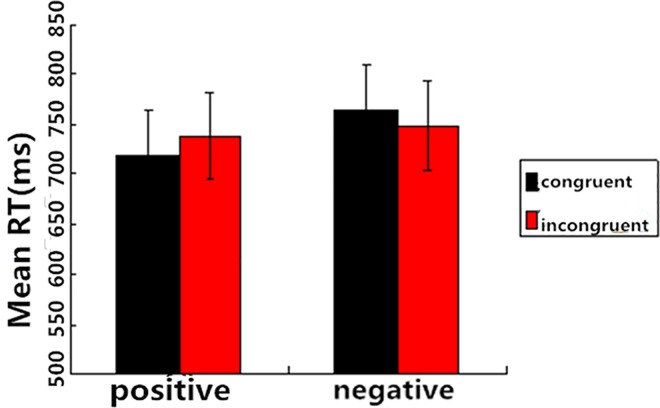
Mean RTs as a function of Prime Congruence and Target Valence (SOA = 200 ms). Red bar refers to negative target and black bar refers to positive target.

We analyzed the RTs for the correct responses to target words because the accuracy in the four conditions was high and it did not differ across conditions. We excluded the RTs above 2000 ms or below 200 ms. A 2 (Prime Condition: incongruent vs. congruent) × 2 (Target Valence: positive vs. negative) repeated-measures ANOVA was performed on the RTs. The main effect of Prime Condition was not significant, *F*(1, 19) = 0.01, *P* > 0.05, *η*^*2*^ = 0.001. However, there was a significant main effect of Target Valence, *F*(1, 19) = 6.34, *P* < 0.05, *η*^*2*^ = 0.25, which indicated that the RTs for positive targets were significantly lower than those for negative targets (729.28 vs. 757.06 ms). More importantly, the interaction between Prime Condition and Target Valence was significant, *F*(1, 19) = 7.04, *P* < 0.05, *η*^*2*^ = 0.27. Post-hoc tests showed that the RTs for positive targets after incongruent primes were significantly shorter than those after congruent primes, *F*(1, 19) = 3.60, *P* = 0.07, and the RTs for negative targets after incongruent primes were longer than those after congruent primes, *F*(1, 19) = 4.54, *P* < 0.05 (Fig 1).

#### ERP results

**Fig 2:**
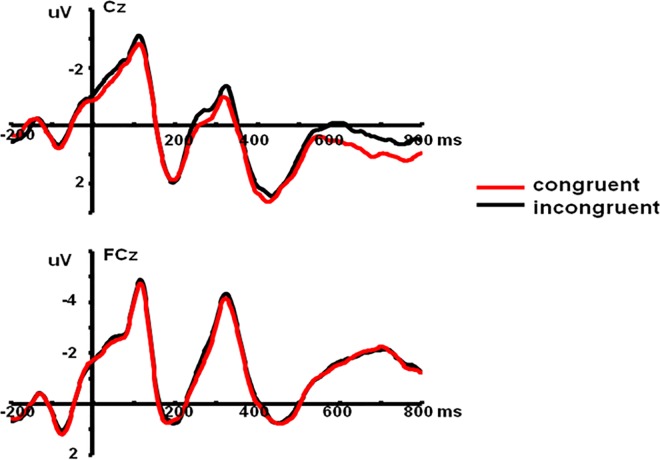
The average ERPs at Cz and FCz for congruent prime and incongruent prime conditions in Stroop effect (SOA = 200ms). Red line represents incongruent prime condition and black line represents congruent prime condition.

We examined the Stroop effect by analyzing the average amplitudes of the N2 component with a 2 (Prime Condition: incongruent vs. congruent) × 10 (Electrode Sites) repeated-measures ANOVA. The main effect of Prime Condition was significant, *F*(1, 19) = 7.80, *P* < 0.05, *η*^*2*^ = 0.29, and the further analyses showed the N2 amplitudes on incongruent primes were larger than those on congruent primes. There was a significant main effect of Electrode Sites, *F*(9, 171) = 4.98, *P* < 0.001, *η*^*2*^ = 0.21, and the interaction between Prime Condition and Electrode Sites was not significant, *F*(9, 171) = 0.78, *P* > 0.05, *η*^*2*^ = 0.04 (Fig 2).

**Fig 3:**
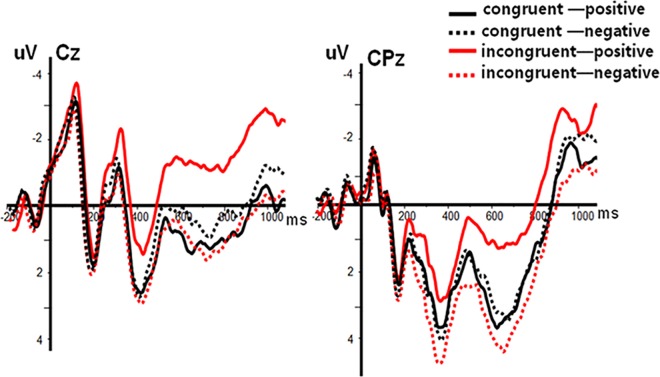
The average ERPs at Cz and CPz for CP (congruent—positive), CN (congruent—negative), IP (incongruent—positive) and IN (incongruent—negative) conditions in priming effect (SOA = 200ms). Red line refers to positive target after congruent prime condition; red dashed line refers to negative target after congruent prime condition; black line refers to positive target after incongruent prime condition and black dashed line refers to negative target after incongruent prime condition.

The affective priming effect was examined by analyzing the average amplitudes of the N400 component with a 2 (Prime Condition: incongruent vs. congruent) × 2 (Target Valence: positive vs. negative) × 9 (Electrode Sites) repeated-measures ANOVA. The results showed that there was a significant main effect of Electrode Sites, *F*(8, 152) = 3.67, *P* < 0.05, *η*^*2*^ = 0.16. However, the main effects of Prime Condition, *F*(1, 19) = 0.65, *P* > 0.05, *η*^*2*^ = 0.03, and Target Valence, *F*(1, 19) = 0.16, *P* > 0.05, *η*^*2*^ = 0.01, were not significant. More importantly, there was a significant interaction between Prime Condition and Target Valence, *F*(1, 19) = 19.98, *P* < 0.001, *η*^*2*^ = 0.51. Subsequent post-hoc analyses showed that the amplitudes of positive targets after congruent primes were significantly lower than those after incongruent primes, *F*(1, 19) = 11.93, *P* < 0.01, while the amplitudes of negative targets after incongruent primes were significantly lower than those after congruent primes, *F*(1, 19) = 4.84, *P* < 0.05. None of the other interactions was significant (all *P*s > 0.05) (Fig 3).

### Discussion

Experiment 1 was designed to demonstrate the negative effect of cognitive conflict processing in the early stage of perception. Therefore, we set the SOA to 200 ms. Results showed that the amplitudes of incongruent primes were higher than those of congruent primes in the Stroop task, revealing a significant conflict effect. More importantly, there was a significant interaction between Prime Condition and Target Valence in the N400 component of the priming effect. The amplitudes of positive targets after congruent primes were significantly lower than those after incongruent primes, and the amplitudes of negative targets after incongruent primes were significantly lower than those after congruent primes, which revealed a negative priming effect at the 200 ms SOA.

The results were consistent with previous studies [13, 15]. They supported the opinion that cognitive conflict was viewed as a negative signal during the early stage of perceptual processing, and subsequently induced a negative priming effect. Generally, once the conflict was detected, it would produce an unpleasant feeling. In addition, the time interval from prime to target was so short that we had no time to adjust the sense of imbalance that could induce a negative influence on subsequent processing.

## Experiment 2

### Methods

#### Participants

Twenty healthy undergraduate students (10 males, 10 females, with average age of 20.9 years, ranging from 19 to 24) volunteered to participate in the study. The rest was similar to the experiment 1.

#### Materials and Procedure

The materials and procedures of experiment 2 were mostly identical to those in experiment 1, except for the following three differences: the valence ratings of the positive targets were over 6.60, the valence ratings of negative targets were below 3.55, and the Stroop primes were presented for 800 ms, so the time window of the N400 should be 1100–1300 ms respectively.

### Results

#### Behavioral results

**Fig 4:**
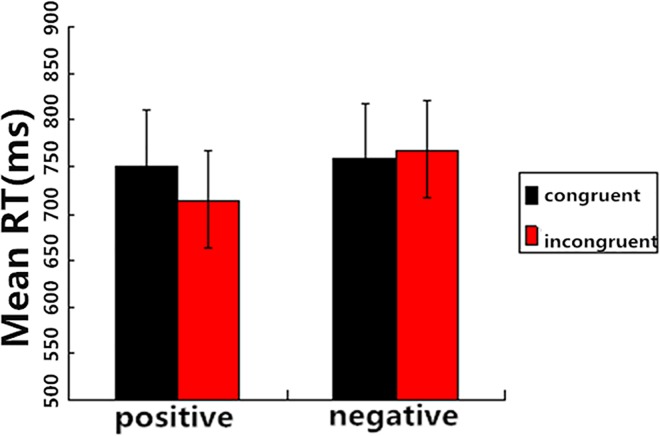
Mean RTs as a function of Prime Congruence and Target Valence (SOA = 800 ms). Red bar refers to negative target and black bar refers to positive target.

The RTs between 200 ms and 2000 ms for correct responses to target words were computed and the rest were excluded from the analyses. A 2 (Prime Condition: incongruent vs. congruent) × 2 (Target Valence: positive vs. negative) repeated-measures ANOVA was performed on the RTs. The main effect of Prime Condition was marginally significant, *F* (1, 19) = 3.28, *P* = 0.09, *η*^*2*^ = 0.15. However, there was a significant main effect of Target Valence, *F*(1, 19) = 6.92, *P* < 0.05, *η*^*2*^ = 0.27, which showed that the RTs for positive targets were significantly lower than those for negative targets (733.17 vs. 764.47 ms). More importantly, the interaction between Prime Condition and Target Valence was significant, *F*(1, 19) = 12.99, *P* < 0.01, *η*^*2*^ = 0.41. Post-hoc tests showed that the RTs for positive targets after incongruent primes were significantly lower than those after congruent primes, *F*(1, 19) = 14.11, *P* < 0.01, while the difference was not significant between the RTs for negative targets after congruent primes and those after incongruent primes, *F*(1, 19) = 0.84, *P* > 0.05 (Fig 4).

#### ERP results

**Fig 5:**
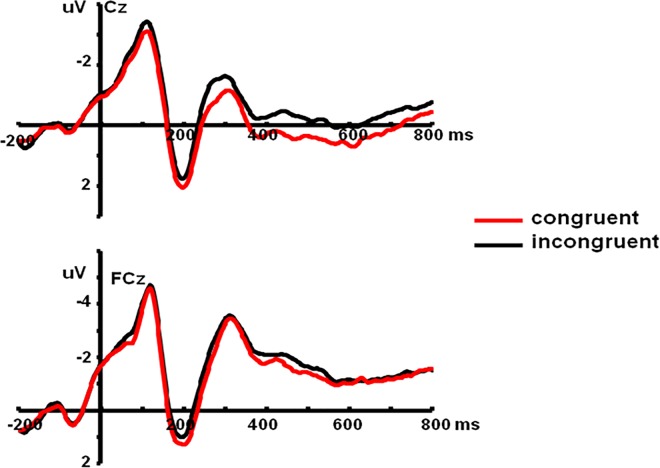
The average ERPs at Cz and FCz for congruent prime and incongruent prime conditions in Stroop effect (SOA = 800ms). Red line represents incongruent prime condition and black line represents congruent prime condition.

The Stroop effect was examined by analyzing the N2 amplitudes using two-factor repeated-measures ANOVA. The main effect of Prime Condition was significant, *F*(1, 19) = 5.74, *P* < 0.05, *η*^*2*^ = 0.23, and the amplitudes of incongruent primes were greater than the amplitudes of the congruent primes. The main effect of Electrode Sites was also significant, *F*(9, 171) = 5.72, *P* < 0.001, *η*^*2*^ = 0.23, but the interaction between Prime Condition and Electrode Sites was not significant, *F*(9, 171) = 1.31, *P* > 0.05, *η*^*2*^ = 0.07 (Fig 5).

**Fig 6:**
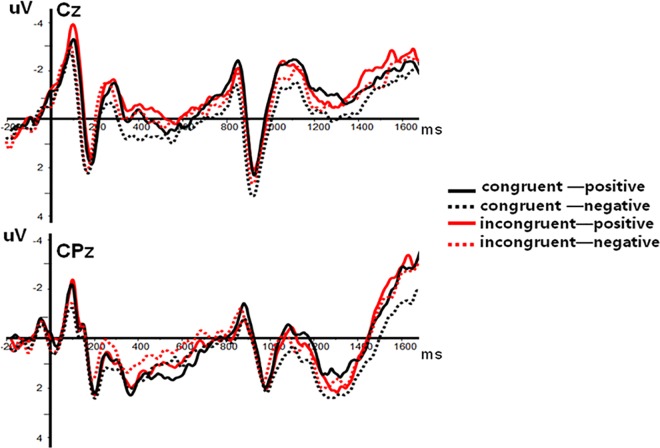
The average ERPs at Cz and CPz for CP (congruent—positive), CN (congruent—negative), IP (incongruent—positive) and IN (incongruent—negative) conditions in priming effect (SOA = 800ms). Red line refers to positive target after congruent prime condition; red dashed line refers to negative target after congruent prime condition; black line refers to positive target after incongruent prime condition and black dashed line refers to negative target after incongruent prime condition.

There was a significant main effect of Electrode Sites in affective priming effect on N400, *F*(8, 152) = 4.16, *P* < 0.001, *η*^*2*^ = 0.18. The main effects of Prime Condition (*F* (1, 19) = 0.40, *P* > 0.05, *η*^*2*^ = 0.02) and Target Valence (*F* (1, 19) = 1.32, *P* > 0.05, *η*^*2*^ = 0.07) were not significant. Crucially, however, the interaction between Prime Condition and Target Valence was significant, *F*(1, 19) = 5.01, *P* < 0.05, *η*^*2*^ = 0.21. Further post-hoc analyses showed that the amplitudes for negative targets after congruent primes were significantly lower than those after incongruent primes, *F*(1, 19) = 5.77, *P* < 0.05, whereas the difference was not significant between the amplitudes for positive targets after incongruent primes and those after congruent primes, *F*(1, 19) = 0.66, *P* > 0.05. None of the other interactions was significant (all *P*s > 0.05) (Fig 6).

### Discussion

The materials and procedure of experiment 2 were mostly identical to experiment 1, excepting one major difference that the time interval from prime to target was set as 800 ms. By doing so, participants had enough time to identify and resolve conflicts. We found that the N2 amplitudes for incongruent primes were greater than those for congruent primes in the Stroop task, which indicated that the Stroop primes really induced cognitive conflict effects. Moreover, in contrast to experiment 1, the results of experiment 2 revealed a positive priming effect that the N400 amplitudes on negative targets after congruent primes were lower than those after incongruent primes.

In line with our hypothesis, when the SOA was 800 ms, there was enough time to identify and resolve conflict. That was to say, we had enough time to adjust the nervousness caused by conflict and then returned to a new balance. Therefore, the conflict effect caused by the Stroop prime was no longer inducing a negative effect on subsequent events and even induced a positive effect.

## General Discussion

The aim of present study was to investigate the function of the signal induced by cognitive conflict during the detection stage or resolution stage. In recent years, a great deal of researches demonstrated the negative nature of cognitive conflict [10, 11, 22, 23, 24], while other researches indicated that the aversiveness of conflict was counteracted by a longer prime presentation, which probably induced positive priming effect [16, 17]. However, the SOAs were all 400 ms in these studies. Since 400 ms might just be the border between the conflict detection and the resolution stage, these previous studies did not really draw any strong conclusions about the priming effect induced by cognitive conflict. Consequently, we used an affective priming paradigm with two opposite SOAs: 200 ms and 800 ms. Cognitive conflict after monitoring (with a SOA of 200 ms) should result in a negative priming effect. Yet, this negative effect would weaken or disappear when the cognitive conflict was resolved (with a SOA of 800 ms) and even the conflict induced a positive priming effect.

Pervious ERPs studies, which aimed to investigate the electrophysiological mechanism of affective priming effect, were concentrated on the N400 components, whose peak was between 350 ms and 550 ms post-stimulus onset. The N400 amplitudes for unrelated prime-target word pairs were more negative than those for related pairs [25], which was the so-called N400 effect. The N400 effect had been observed in various studies that larger N400 amplitude induced by affectively incongruent trials compared to congruent trials [26, 27, 28]. In the studies of affective priming, affective primes incompatible with targets would gave rise to N400 effect. Another study found that word pairs produced the N400 even when the following word had nothing to do with the preceding word [29]. The automatic activation of prime stimuli appeared to lead to the pre-activation of target stimuli and the declining on the N400 amplitudes. If the target stimuli were not pre-activated by prime stimuli, the N400 amplitudes were larger correspondingly.

The present results confirmed our hypotheses. When the SOA was 200 ms that probably meant conflict was at the detection stage, the N400 amplitudes of negative targets after incongruent primes were significantly lower than those after congruent primes, which showed a significant negative priming effect. However, the N400 amplitudes showed a significant positive priming effect when the SOA extended to 800 ms that predicted conflict might be at the resolution stage.

Because participants in this study did not need to do behavioral response to the Stroop primes, it could not be completely ensured whether the subjects viewed the Stroop stimuli all the time, although we had arranged the catch trials. However, Botvinick [2] said that conflict also might be triggered by any “simultaneous activation of incompatible representations.” Furthermore, van Veen et al. [30] stated that conflict monitoring was not restricted by the response level. Therefore, we could infer that the Stroop primes induced the following priming effect on the EEG data.

In conclusion, cognitive conflicts could be viewed as negative evaluative signals during the detection stage, and induced negative priming effects on subsequent cognitive processing. Once the conflict was resolved (that is, the processing of the conflict was completed), the negative effect would weaken or even disappear. Thus, it no longer had negative effect on the subsequent processing, and even induced positive effect.

## Supporting Information

S1 Dataset Behavior data. (XLS)

S2 Dataset ERP data. (XLS)
